# Agression and violent behaviour risk assessment

**DOI:** 10.1192/j.eurpsy.2021.1013

**Published:** 2021-08-13

**Authors:** G. Marinho, S. Almeida

**Affiliations:** 1 Clinica 6, CHPL, Lisbon, Portugal; 2 Serviço De Psiquiatria, Hospital Prisional S. João Deus, Lisboa, Portugal

**Keywords:** violence, risk assessment, forensic psychiatry

## Abstract

**Introduction:**

After discharge from forensic psychiatric hospital, rates of violent reoffending are reported to range from 2% to 8% per year in high income countries. Risk assessment informs decisions around admission to and discharge from secure psychiatric hospital and contributes to treatment and supervision Current approaches to assess violence risk in secure hospitals are resource intensive, limited by accuracy and authorship bias. Forensic Violence Oxford (FoVOx) was developed using all forensic psychiatric patients in Sweden, based on the largest forensic psychiatric sample to date, and has the advantage of using routinely available data, which are less liable to bias than interview-based measures.

**Objectives:**

Literature review on the Forensic Psychiatry and Violence Oxford (FoVOx) tool.

**Methods:**

Pubmed and Google Scholar search

**Results:**

The 12 items within the FoVOx tool are sex, age, previous violent crime, previous serious violent crime, primary discharge diagnosis, drug use disorder at point of hospitalization or discharge, any lifetime drug use disorder, alcohol use disorder at point of hospitalization or discharge, personality disorder at discharge, employment at admission, five or more prior inpatient episodes, and whether current length of stay has exceeded one year.

**Conclusions:**

The FoVOx tool is scalable, quick, free to use and available online. Its use could enable clinicians to provide a reasonably accurate risk assessment in a brief and cost-effective way, and free up time to focus on clinical care and risk management rather than risk assessment.

